# Dosimetric sensitivity of an enhanced leaf model (ELM) for individual versus averaged machines

**DOI:** 10.1002/acm2.14621

**Published:** 2025-02-04

**Authors:** Rafail Panagi, Rhydian Caines, Carl G. Rowbottom

**Affiliations:** ^1^ Medical Physics Department The Clatterbridge Cancer Centre NHS Foundation Trust Liverpool UK

**Keywords:** dosimetric leaf gap, enhanced leaf modeling, multi‐leaf collimator

## Abstract

**Background:**

With the introduction of a new multi‐leaf collimator (MLC) enhanced leaf model (ELM) in the Varian Eclipse™ treatment planning system, there is currently limited data regarding the dosimetric sensitivity to real‐world variation in the ELM parameters, and its clinical relevance.

**Purpose:**

To characterize the variation in ELM parameters across a large department with ten linear accelerators and investigate the feasibility of using a single machine‐averaged ELM for treatment planning. This could achieve time and resource savings from reduced quality assurance, while allowing easy transfer of patients between machines.

**Methods:**

Clinical plans of a range of sites (head and neck, prostate, breast, lung, and brain), techniques (VMAT, IMRT, SBRT, and SRS), and energies (6 MV, 6 MV FFF, 10 MV, and 10 MV FFF) were recalculated on Varian TrueBeam™ (120 MLC) and Varian EDGE™ (HD120 MLC), with machine‐specific ELM beam models, an averaged machine and an outlier machine model. A range of clinically relevant metrics relating to target coverage (e.g. PTV D_98%_, D_50%_, D_2%_) and OAR doses (dosimetric, volumetric, conformity, and gradient indices) were evaluated.

**Results:**

For the target metrics, the maximum percentage deviation from the mean was 0.422%, 0.157%, and 1.956% for the cases of the individual machines, the averaged machine and the outlier machine correspondingly, while the maximum absolute dose differences were 0.28 Gy, 0.07 Gy, and 0.38 Gy. For the OAR metrics, the maximum deviation from the mean was 1.833%, 0.204%, and 5.722% for the individual, averaged, and outlier machines, while the maximum absolute dose differences were 0.41 Gy, 0.10 Gy, and 0.97 Gy.

**Conclusions:**

For machines that are well matched in terms of dosimetry for transmission and sweeping gap fields, the use of an averaged machine model is unlikely to introduce clinically significant dosimetric differences to treatment plans.

## 1 INTRODUCTION

Positional and dosimetric accuracy is of the utmost importance in radiotherapy, and the treatment planning system (TPS) beam and collimation models are important sources of uncertainty. An important element of the photon fluence model is the multi‐leaf collimator (MLC). With the aim of improving accuracy, one vendor (Varian Medical Systems, Palo Alto, California) has recently introduced a more sophisticated MLC model in the latest version of their TPS, Eclipse (v18.0), termed an enhanced leaf model (ELM).

A previous Eclipse MLC model made some simplifying assumptions. First, the MLC rounded leaf tips were assumed flat, and an offset to the nominal leaf position was introduced for fluence calculation to simulate the effect of partial leaf tip transmission on the beam penumbra.^1^ This offset, known as dosimetric leaf gap (DLG), approximates the situation of curved leaf ends that remain dosimetrically partially “open,” even when they are mechanically “closed.” DLG would be determined by the end user by extrapolation of measured dose across a series of sweeping gap test fields.^1^ Adjustments to the beam model target spot size might then be needed to offset inaccuracies in beam penumbra arising from this approach.^2^, ^3^ Furthermore, MLCs were treated as thin homogeneous layers that attenuated the fluence when in the beam by a linear transmission factor (LTF). This approach assumed a constant LTF value for different leaf gauges within a carriage, and averaged the effect from the presence of the drive‐screw cutout.

Thus, the whole MLC model was previously characterized by two empirical parameters, introducing uncertainty, particularly in the case of small off‐axis openings where fluence could be misrepresented as a result of these simplifying assumptions. In particular, the concept of a thin attenuating layer meant differences in oblique path length through the MLC and its tip was not accounted for as an aperture moves across a field. Fluence was dependent on the size of a field opening, but not its position relative to the central axis.

**FIGURE 1:**
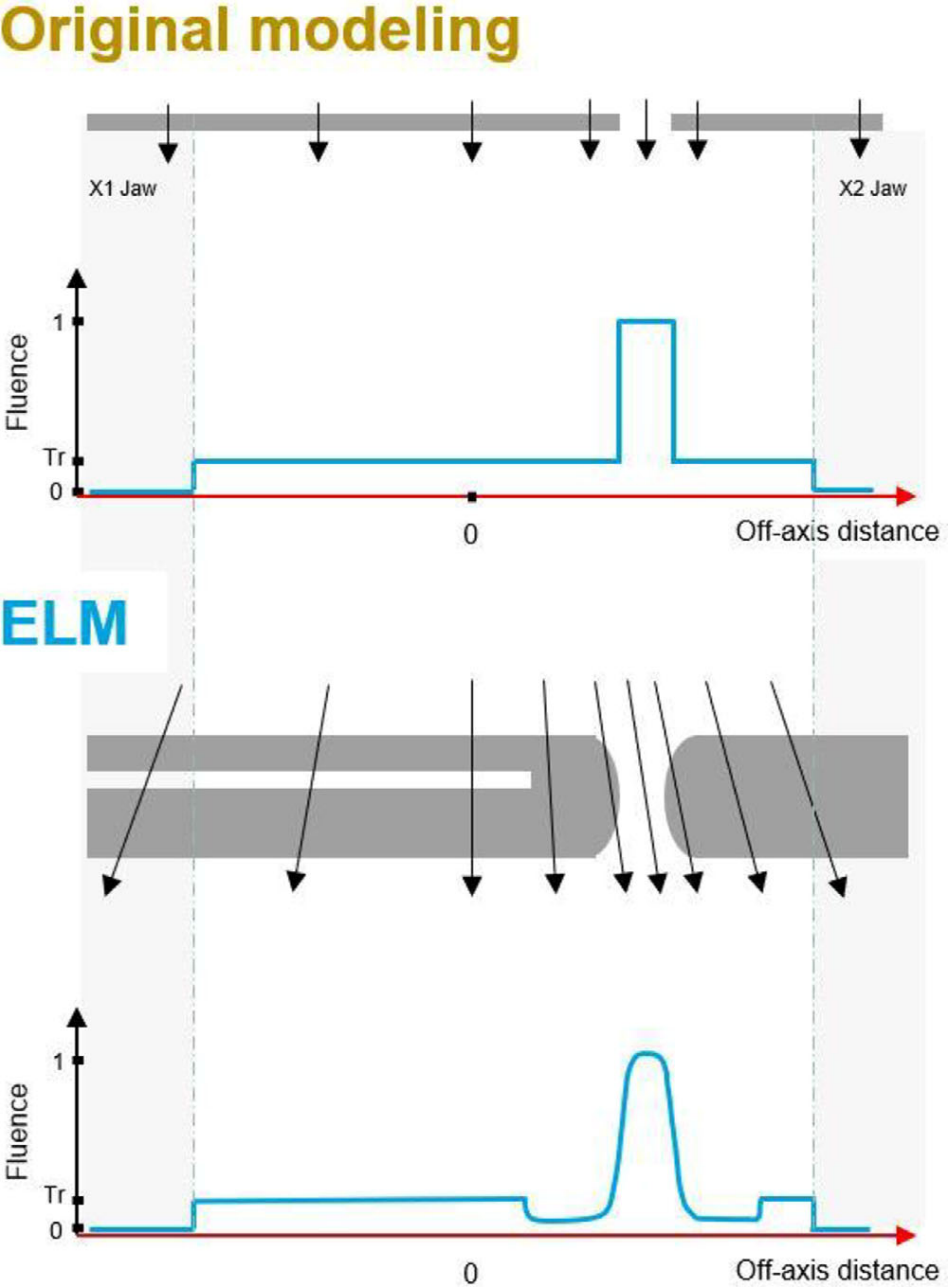
Original versus enhanced leaf model. The original MLC model and the assumptions made resulted in a more simplified fluence profile, along with the ELM and the improvements made in the modeling of the fluence. Reproduced with permission from Varian and Ann Van Esch. ELM, enhanced leaf model; MLC, multi‐leaf collimator.

The ELM offers a more sophisticated representation of the shape of the MLC that seeks to improve dosimetric accuracy. As it has been recently described by Van Esch et al.,^4^ the ELM explicitly models the rounded leaf tip, the drive screw cut‐out, and the MLC thickness.^5^ As before, parameters for the ELM are fit to user measurements to a set of open, closed, and sweeping gap fields. However, this is now done by an internal process of parameter fitting to match a set of measured relative charge values, rather than entering the empirically derived values of DLG and LTF. The original MLC model and the ELM and their effect on fluence along the direction of MLC motion are illustrated in Figure 1.

**FIGURE 2:**
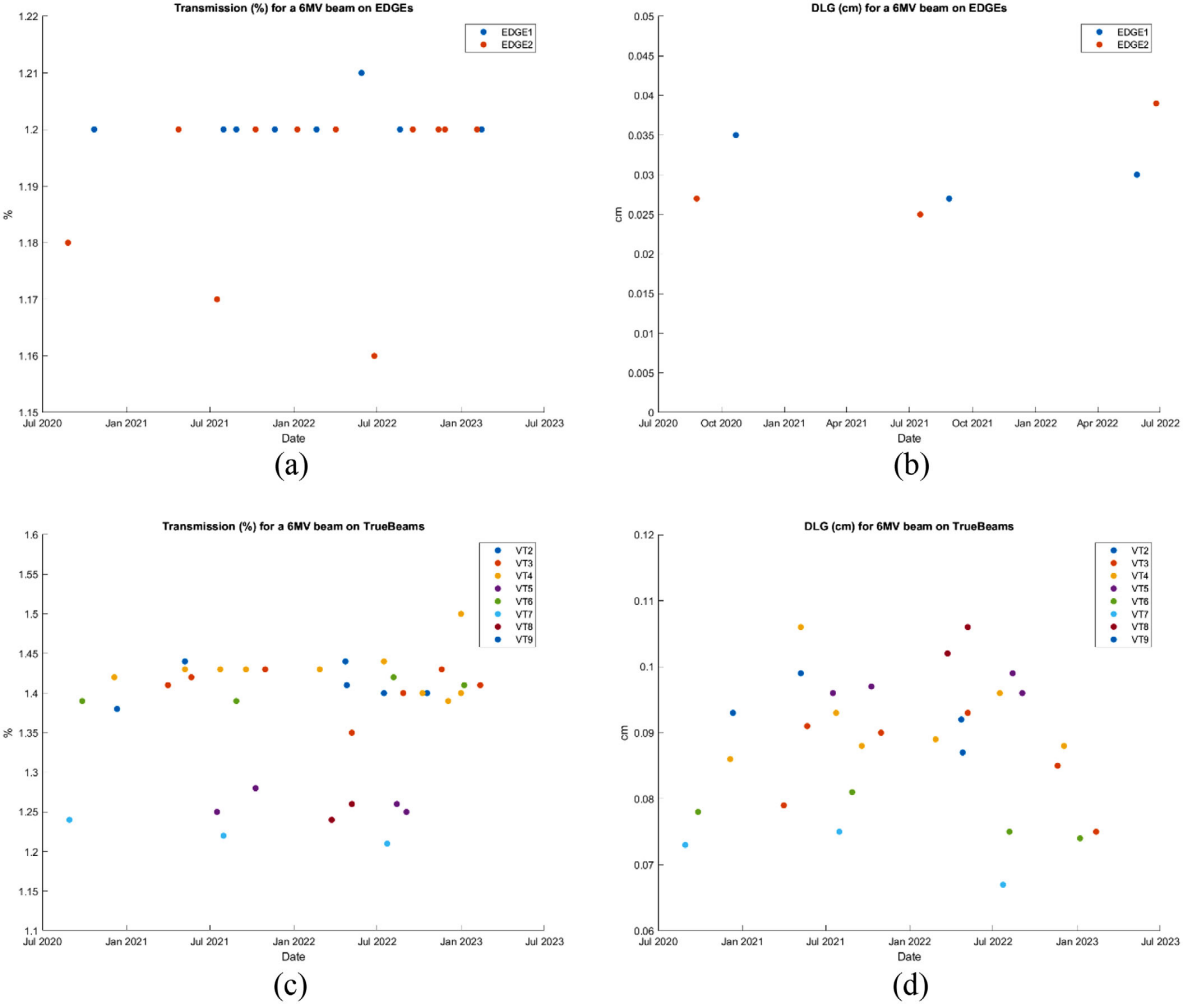
Audit of DLG and LTF for our eight Varian TrueBeam™ and two Varian EDGE™ machines. Results of a local audit of LTF and DLG for our two Varian EDGE™ machines (a) and (b) and the eight Varian TrueBeam™ (c) and (d) showing the spread of values over a period of time from July 2020 to July 2023. DLG, dosimetric leaf gap; LTF, linear transmission factor.

The dosimetric effect of variations in the original MLC model has been evidenced.^6^ However, for the ELM, there is limited understanding of dosimetric sensitivity to input parameters. Having a single beam and MLC model is operationally desirable as it allows easier transfer of patients between machines, without the need for dose recalculation. Especially, when the spread of DLG and LTF values is relatively small (see Figure 2 and Table 1).

**TABLE 1:** DLG and LTF values.

	DLG (cm)		LTF (%)	
	VT	EDGE	VT	EDGE
6 MV	0.067–0.106	0.025–0.039	1.21–1.50	1.16–1.21
6 MV FFF	‒	0.017–0.030	‒	0.96–1.01
10 MV	0.086–0.123	0.035–0.048	1.46–1.73	1.35–1.44
10 MV FFF	0.073–0.112	‒	1.29–1.52	‒

*Note*: The range of values for all dosimetric leaf gaps (DLG) and linear transmission factors (LTF) for both types of machines/MLC model and all energies in our centre. Missing values indicate energies that are not commissioned clinically.

In this study, we aim to develop an understanding of real‐world ELM sensitivity in configuration values by exploiting the existing dosimetric variation within our local linac fleet. In particular, we wanted to understand whether for common, clinically representative plan types, there was a clinically or dosimetrically significant difference between machine‐specific or machine‐averaged ELMs. Accepting our own linacs are already relatively well matched, we further created some “outlier” models by simulating a higher level of variation between our machines. Finally, we seek to address the operational question of the feasibility of a machine‐averaged ELM in these common clinical scenarios. It is hoped this information will be useful to other centers who may be commissioning their own ELMs in the future.

## 2 METHODS

### 2.1 MLC configuration

Measurements of open field, transmission, and sweeping gaps on each machine were used to produce ten machine‐specific ELM models: Eight Varian TrueBeam™ (VT2, VT3… VT9) with Millennium 120 MLCs (m120) and two Varian EDGE™ machines (EDGE1 and EDGE2) with HD120 MLCs were used in this study across three geographical sites. All measurements were made using a type NE2571A 0.6cc Farmer chamber in a water phantom at 10 cm depth, with a SSD of 90 cm. An “averaged” ELM model was also generated for the TrueBeam (VT) and EDGE (VED) machine types respectively. Within each type, the charge values were averaged and configured as separate machines, respectively designated “VT” and “VED” in the Eclipse Beam Configuration workspace. The charge values of these averaged machines along with standard deviations are shown in Tables 2 and 3 to demonstrate the measured range.

**TABLE 2:** Average of field charge measurements for TrueBeam linacs.

	6×		10×		10FFF	
	Average (nC)	Std. dev. (nC)	Average (nC)	Std. dev. (nC)	Average (nC)	Std. dev. (nC)
Open field	17.579	0.236	19.621	0.431	18.580	0.209
Transmission A	0.245	0.018	0.312	0.028	0.269	0.020
Transmission B	0.247	0.018	0.319	0.024	0.271	0.020
Sweeping gap 4 mm	0.966	0.030	1.148	0.036	1.042	0.029
Sweeping gap 6 mm	1.243	0.031	1.458	0.036	1.335	0.029
Sweeping gap 20 mm	3.265	0.053	3.718	0.057	3.473	0.045

*Note*: Averages and standard deviations of measurements for open field, transmission across each jaw bank (A and B), and sweeping gaps of all eight TrueBeam™ machines with m120 MLC.

**TABLE 3:** Average of field charge measurements for EDGE linacs.

	6×		10×		6FFF	
	Average (nC)	Std. dev. (nC)	Average (nC)	Std. dev. (nC)	Average (nC)	Std. dev. (nC)
Open field	17.385	0.080	19.555	0.023	16.322	0.011
Transmission A	0.219	0.010	0.277	0.011	0.175	0.009
Transmission B	0.216	0.003	0.274	0.003	0.173	0.004
Sweeping gap 4 mm	0.842	0.003	0.991	0.009	0.751	0.005
Sweeping gap 6 mm	1.116	<0.001	1.299	0.007	1.009	0.004
Sweeping gap 20 mm	3.120	0.009	3.547	0.010	2.894	0.003

*Note*: Averages and standard deviations of measurements for open field, transmission across each jaw bank (A and B), and sweeping gaps of the two EDGE™ machines with HD120 MLC.

Lastly, “outlier” machines “VT+” and “EDGE+” were contrived by simulating a very large variation between existing machines. For VT+ five standard deviations were added to each of the sweeping gap and LTF values, to simulate the spread of DLGs noted in the study by Glenn et al.^7^ Finding large data samples from HD120 MLC for the 6 MV FFF energy used for SRS, proved more difficult. In order to simulate a similar spread to the VT+ model, a much larger number of standard deviations needed to be added due to how well matched the EDGE1 and EDGE2 machines are in this study's cohort. The required range of standard deviations (for sweeping gap and LTF) agreed well with a smaller sample of data published by Saez et al.^8^ for 6 MV. An assumption is made here that the spread for 6 MV and 6 MV FFF would be comparable. The average was taken and 25 standard deviations were added to the data to simulate the VED+ model.

### 2.2 Clinical plan selection

Clinical plans from a variety of sites and energies were selected and re‐calculated for each relevant machine‐specific, averaged, and outlier ELM models, using the local clinical normalization and keeping all other parameters constant. The sample of clinical plans used in this study consisted of: 10 Head and Neck 6 MV VMAT, 10 Prostate 6 MV VMAT, 5 Breast 6 MV sliding window IMRT, 5 Breast 10 MV sliding window IMRT, 5 Breast 10XFFF sliding window IMRT, 10 Lung 10 MV FFF SBRT and 10 Brain multi‐metastases (≥ 2 targets) 6 MV SRS. The SRS Brain multi‐metastases are planned on machines using the HD120 MLC, the rest are planned using the 120 MLC. For plans using 10 MV FFF, only six of our eight VT machines were used as the 10FFF energy is not commissioned on two of them. With the exception of the Lung 10 MV FFF SBRT plans, all plans used in this study had previously been used clinically at our center and subject to standard clinical verification and quality assurance (QA).

For each plan type and site, site‐specific clinically relevant metrics were selected to survey the differences observed in target coverage and OARs, see Table 4. Since a normalization was applied in each case, MU for each calculation was also recorded.

**TABLE 4:** Metrics used for each site.

Plan type	Target coverage metrics	OAR metrics	Normalization method and metric	No. of ELM variations	No. of patients
H&N VMAT	PTV D_98%_, D_50%_, and D_2%_	SpinalCanal D_0.1cc,_ Parotid L/R D_mean,_ Larynx D_mean_	Target Mean (MU/Gy)	10	10
Prostate VMAT	PTV D_98%_, D_50%_, and D_2%_	Rectum V_52Gy_ and V_32Gy,_ Bladder V_48Gy_	Target Mean (MU/Gy)	10	10
Breast IMRT	CTV D_98%_, D_50%_, and D_2%_	Ipsilateral Lung V_30Gy_	Target Mean (MU/Gy)	10 8 for 10FFF	15
Lung SBRT	PTV D_99%_ and D_0.5%_	PTV + 2 cm D_0.1cc,_ Lungs‐IGTV V_20Gy_	D_95%_ = 100% (MU/Gy)	8	10
SRS multi‐metastases	PTV D_max_ and D_min_	Paddick CI & GI, Brain‐PTV V_12Gy_	None	4	10

*Note*: Reported metrics for target coverage and OAR doses with normalization method and relevant metric. The number of patients and different ELM variation used to recalculate are also specified, these include the averaged and outlier ELM model.

Metrics of absolute dose were normalized as a percentage to the median of all machines while metrics with units of percentage were normalized as a difference to the median of all machines.

## 3 RESULTS

**FIGURE 3:**
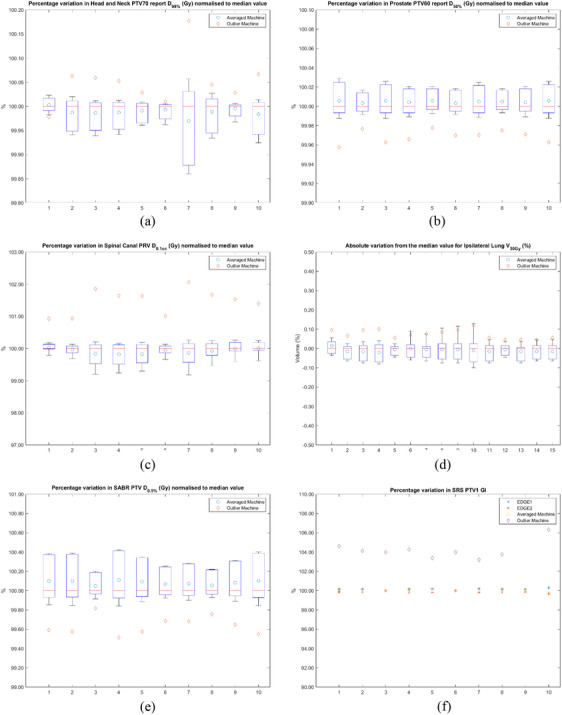
Box plots for a representative selection of metrics across all five sites. Examples of box plots demonstrating the variation in the metrics across all machines for each patient for all sites. PTV D_98%_ for VMAT H&N plans (a), PTV D_50%_ for VMAT prostate plans (b), spinal canal PRV D_0.1cc_ for H&N plans (c), ipsilateral lung V_30Gy_ for FF IMRT breast plans (d), PTV D_0.5%_ for SBRT lung plans (e) and GI for SRS plans (f).

A representative sample of the produced plots including target and OAR metrics from all sites can be seen in Figure 3. The spread of values from the individual machines can be seen in the boxplots, while the averaged and the outlier machines are denoted separately. Due to only having two HD120 MLC machines, the two individual machine models, the averaged and the outlier machine are all denoted individually in the case of SRS brain multi‐metastases plots.

Maximum deviations of individual machines from the median value for target and OAR metrics and MU/Gy for each site can be seen in Table 5. Similarly, maximum deviation from the median for the averaged machine and the outlier machine can be seen in Tables 6 and 7 correspondingly.

**TABLE 5:** Maximum deviations of all individual machines from the median values.

	Target	OAR	MU/Gy	CI/GI
H&N	0.140% (0.09 Gy)	1.833% (0.37 Gy)	0.757% (1.90)	‒
Prostate	0.166% (0.10 Gy)	0.410% (0.41 Gy)	0.525% (3.15)	‒
Breast	0.134% (0.04 Gy)	0.130% (0.13 Gy)	0.508% (1.38)	‒
SABR lung	0.422% (0.28 Gy)	0.329% (0.09 Gy)	2.392% (4.45)	‒
SRS	0.165% (0.04 Gy)	0.254% (0.04 Gy)	‒	0.415% (0.03)

*Note*: Maximum deviation of all individual machines from the median value for metrics related to target coverage, OAR doses, and MU/Gy for each site and CI/GI for the multi‐met SRS patients.

**TABLE 6:** Maximum deviation of the averaged machine from the median values.

	Target	OAR	MU/Gy	CI/GI
H&N	0.030% (0.02 Gy)	0.204% (0.09 Gy)	0.159% (0.40)	‒
Prostate	0.038% (0.02 Gy)	0.100% (0.10 Gy)	0.171% (0.75)	‒
Breast	0.058% (0.02 Gy)	0.020% (0.02 Gy)	0.149% (0.37)	‒
SABR lung	0.112% (0.07 Gy)	0.067% (0.05 Gy)	0.200% (0.46)	‒
SRS	0.157% (0.04 Gy)	0.141% (0.02 Gy)	‒	0.301% (0.01)

*Note*: Maximum deviation of the averaged machine from the median value for metrics related to target coverage, OAR doses, and MU/Gy for each site and CI/GI for the multi‐met SRS patients.

**TABLE 7:** Maximum deviation of the outlier machine from the median values.

	Target	OAR	MU/Gy	CI/GI
H&N	0.177% (0.12 Gy)	5.363% (0.97 Gy)	1.614% (4.05)	‒
Prostate	0.244% (0.14 Gy)	0.755% (0.75 Gy)	0.930% (5.65)	‒
Breast	0.166% (0.04 Gy)	0.120% (0.12 Gy)	1.056% (2.83)	‒
SABR lung	0.485% (0.32 Gy)	0.657% (0.16 Gy)	0.945% (2.20)	‒
SRS	1.956% (0.38 Gy)	5.722% (0.94 Gy)	‒	7.779% (1.72)

*Note*: Maximum deviation of the outlier machine from the median value for metrics related to target coverage, OAR doses, and MU/Gy for each site and CI/GI for the multi‐met SRS patients.

**FIGURE 4:**
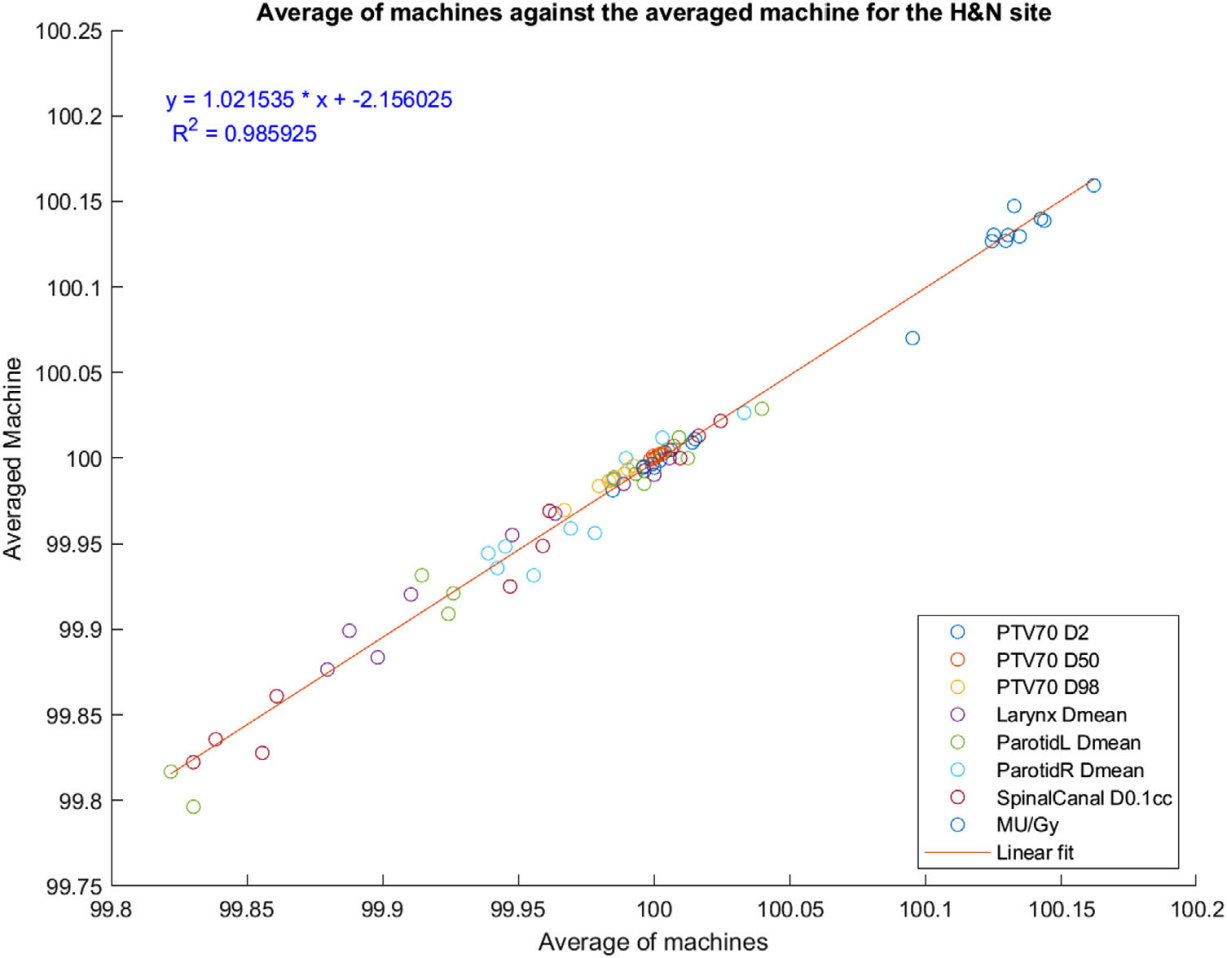
Comparison of averaged Varian TrueBeam™ machine versus the average of all Varian TrueBeam™ machines. A comparison between the averaged Varian TrueBeam™ machine and the average of the eight Varian TrueBeam™ machines for all variables for the H&N plans, along with a linear fit showing a good fit to the data.

To demonstrate the suitability of using an averaged machine model for all our linacs, a comparison of all the variables of the averaged machine to the average of variables of each machine‐specific model was made. The results for the H&N site are plotted in Figure 4, the other sites demonstrated similar results. As shown by the linear fit, the averaged machine model is a suitable representation of the average of all machines for H&N plans.

## 4 DISCUSSION

It becomes evident that for well‐matched machines, such as those in our department, where legacy DLG and LTF variation is bounded within the values cited in Table 1, differences arising from the various ELM models are not clinically significant. This is shown for a range of common sites and varying complexity. For all sites, the maximum percentage deviation of any machine from the median value in target coverage variables was 0.422%, for OAR doses it was 1.833%, for MU/Gy it was 2.392% and for conformity and gradient indices, it was 0.415%. The maximum absolute value deviations were 0.28 Gy, 0.41 Gy, 4.45 MU/Gy, and 0.03 for the target coverage, OAR doses, MU/Gy and conformity and gradient indices respectively. The deviations noted for the case of the individual machines are well within or of the same order (2%–3%) of other uncertainties introduced in the radiotherapy pathway, and the dose deviations are unlikely to have a clinically significant effect on the plan.

In the case of the outlier machine, the maximum percentage difference from the median across all sites was 1.956%, 5.722%, 1.614%, and 7.779% for target coverage, OAR doses, MU/Gy, and conformity and gradient indices respectively. The maximum absolute differences were 0.38 Gy, 0.97 Gy, 5.65 MU/Gy, and 1.72. These deviations are larger and could potentially have a clinically significant effect, especially in the case of OAR doses where they could surpass specified tolerances. This is in agreement with results for the legacy MLC model, by Glenn et al.,^9^ which demonstrated that for machines employing outlier LTF and DLG values, the dosimetric effect could be clinically significant. The study reported dosimetric differences in the target of up to 6% and to OARs of up to 10%.^9^ A later study showed that centers with atypical MLC model parameters were more likely to fail dose delivery audits.^10^ The dosimetric effect calculated in this study appears to be less significant than the one reported from studies on the legacy model. Even in the case of the more extreme outlier model used in this study, dose variation was less than 1.956% in the target and less than 5.722% in OARs.

The outlier models used in this study were artificially created to simulate the outlier results of the aforementioned audit. In particular, the same simulated differences were used in both sweeping gaps and LTF measurements. This likely represents a higher than reasonably expected variation in LTF, which unlike DLG is not subject to variation in the mechanical leaf calibration. Nonetheless, these outlier models indicate a valid upper bound to the dosimetric differences that could be expected, and the reported results are comparable with or lower than those previously observed.

The averaged machine concept proves to be a good representation of the range of values of individual machines as the maximum deviation from the median for all machines was 0.157%, 0.204%, 0.200%, and 0.301% for target coverage, OAR doses, MU/Gy and conformity and gradient indices respectively. The absolute maximum differences were 0.07 Gy, 0.10 Gy, 0.75 MU/Gy, and 0.01 for the target coverage, OAR dose, MU/Gy normalization, and conformity and gradient indices. This indicates for other centers that, provided legacy DLG and LTF variation is comparable to the present study, a single averaged ELM is a feasible proposition. This in turn enables time and resource efficiencies from reduced TPS QA and easier patient transfers.

When examining the case of the outlier machines, the SRS site planned using the HD120 MLC demonstrates a typically larger variation from the median value when compared to other sites planned with the m120. While noting MU/Gy were typically higher in these HD120 plans, this is nonetheless consistent with Van Esch et al.^4^ who showed the ELM had the greatest impact in the case of small, off‐axis openings—precisely as found in these SRS‐type multi‐target plans. This is also in line with results published by Saez et al.^8^ regarding differences in TPS calculated doses for DLGs derived in different ways from either synchronous or asynchronous sweeping gap plans, for the legacy MLC model. For Varian linacs, using the HD120 MLC model differences of more than 10% were demonstrated, while the m120 model demonstrated differences of about 5%. However, the fields used were artificial and designed to maximally express differences in MLC modeling, especially by exaggerating the tongue‐and‐groove effect.^8^

There are a few limitations to note. First, the results rely on a set of machines that are very well matched with small standard deviations for open field, LTF, and sweeping gap measurements (Tables 2 and 3). The effect of an outlier machine was demonstrated artificially (albeit with an unrealistically large LTF). Nonetheless, this artificial construct retains value in demonstrating a reasonable upper limit to the plan variation that might be contributed by the ELM. Indeed, even in this extreme scenario many of the clinically relevant parameters appeared negligibly perturbed. Second, all data collected were based on locally produced plans, many of which had a plan robustness MU limitation at 250–260 MU/Gy, inherently limiting the ELM impact. Caution is advised when extrapolating these results to plans with significantly higher modulation. Ideally, a larger number of treatment sites and patients should be used; however, this study was limited in resources and time. As it can be seen in Figure 3 there seems to be greater patient sensitivity than model sensitivity in some cases. This could be due to specific properties of individual plans, such as aperture shape and size across all control points. Lastly, the scope of this study was limited to locally accessible traditional C‐arm Varian TrueBeam™ linear accelerators. However, Halcyon and other O‐ring‐based treatment delivery systems that are emerging as an important modality for many radiotherapy applications, include the use of double‐stacked MLCs, and may themselves be subject to important inter‐machine variations, which have not been characterized here.

## 5 CONCLUSION

This study highlighted the MLC ELM dosimetric sensitivity in treatment plans of varying complexity for several clinically relevant sites. Centers looking to commission their own ELMs may wish first to audit their own variation in legacy DLG and LTF measurements. Where such variation is comparable to that reported in the present study, it is feasible that a single averaged ELM will have negligible clinical impact on calculated treatment plans. This study therefore potentially offers a basis for decision‐making regarding the creation and handling of ELM in such situations. In closing, these findings contribute to the ongoing work of understanding the new ELM performance and its ramifications in clinically relevant circumstances.

## AUTHOR CONTRIBUTIONS

The authors confirm their contribution to the paper as follows: *Study conception and design*: Rhydian Caines, Rafail Panagi, and Carl Rowbottom. *Data collection*: Rafail Panagi and Rhydian Caines. *Analysis and interpretation of results*: Rafail Panagi and Rhydian Caines. *Draft manuscript preparation*: Rafail Panagi and Rhydian Caines. *Critical review of manuscript*: Carl Rowbottom. All authors reviewed the results and approved the final version of the manuscript.

## CONFLICT OF INTEREST STATEMENT

The Clatterbridge Cancer Centre is a Varian reference site and participated in the Limited Launch Programme (LLP) for Eclipse v18.0. The authors have no affiliations or financial interest.
